# Peripheral perfusion index measured using magnetohydrodynamic voltages in 3T MRI

**DOI:** 10.1186/1532-429X-16-S1-P156

**Published:** 2014-01-16

**Authors:** Thomas S Gregory, Ehud J Schmidt, Shelley H Zhang, Jonathan R Murrow, Zion T Tse

**Affiliations:** 1 College of Engineering, University of Georgia, Athens, Georgia, USA; 2 Brigham and Women's Hospital, Boston, Massachusetts, USA; 3 GRU-UGA Medical Partnership, University of Georgia, Athens, Georgia, USA

## Background

The Peripheral Perfusion Index (PFI) has been utilized for early detection of impaired organ perfusion in order to avoid tissue hypoxia, which could lead to organ failure [1]. A decrease in effective circulating blood volume, lowering of PFI levels, can cause vasoconstriction [2]. Strong MRI magnetic field (B0) interactions with flowing blood plasma electrolytes produce a Magnetohydrodynamic voltage (VMHD) [3]. We hypothesized that a processing method which derives VMHD at different segments of the body could provide a direct indicator for PFI as well as local perfusion levels in various body regions. Existing methods for PFI estimation include Pulse Oximetry (PO) and differential temperature recordings, both of which are indirect measurements [4].

## Methods

**Figure 1:**
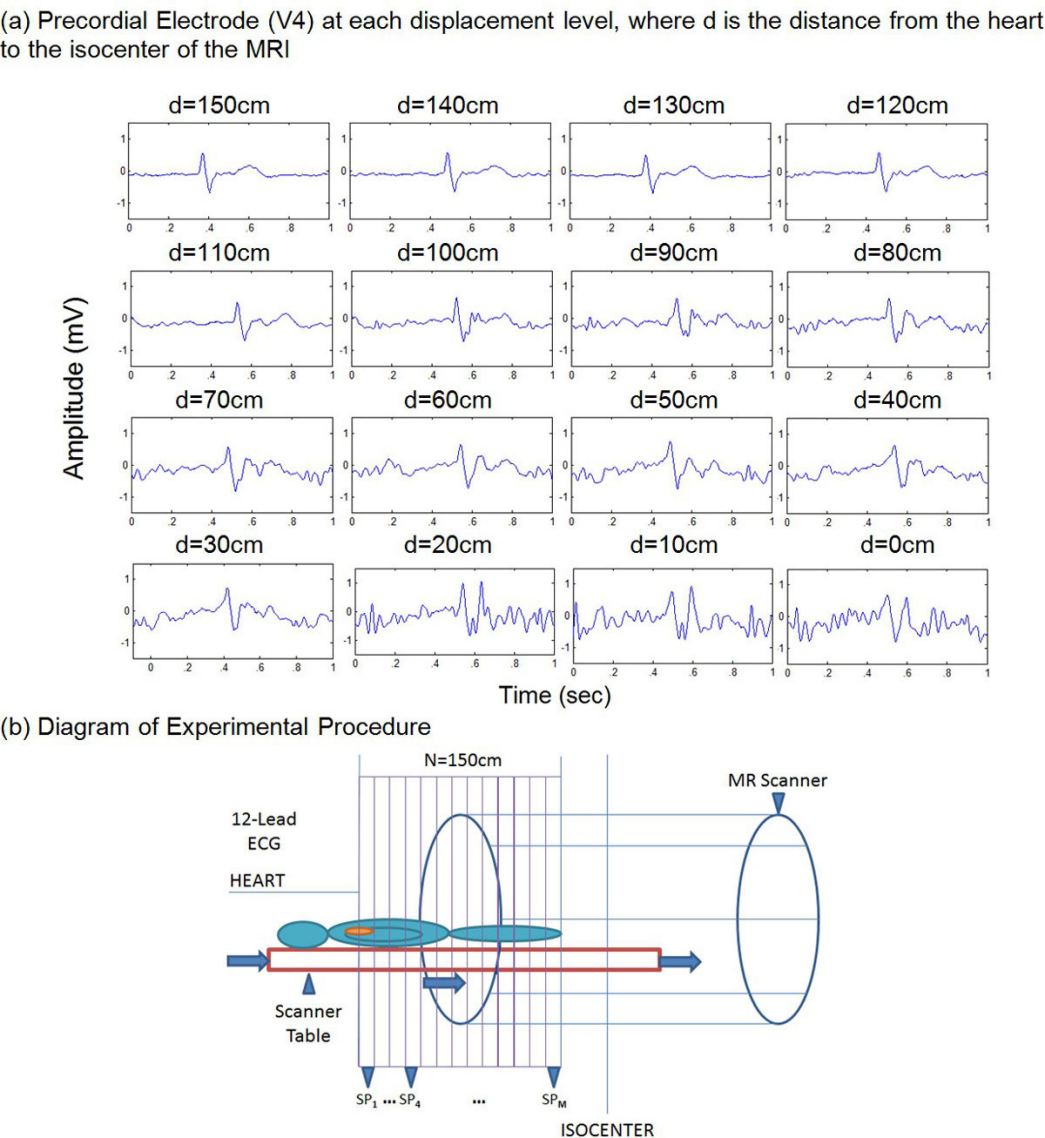
**Recording of raw data for MHD perfusion mapping**.

**Figure 2:**
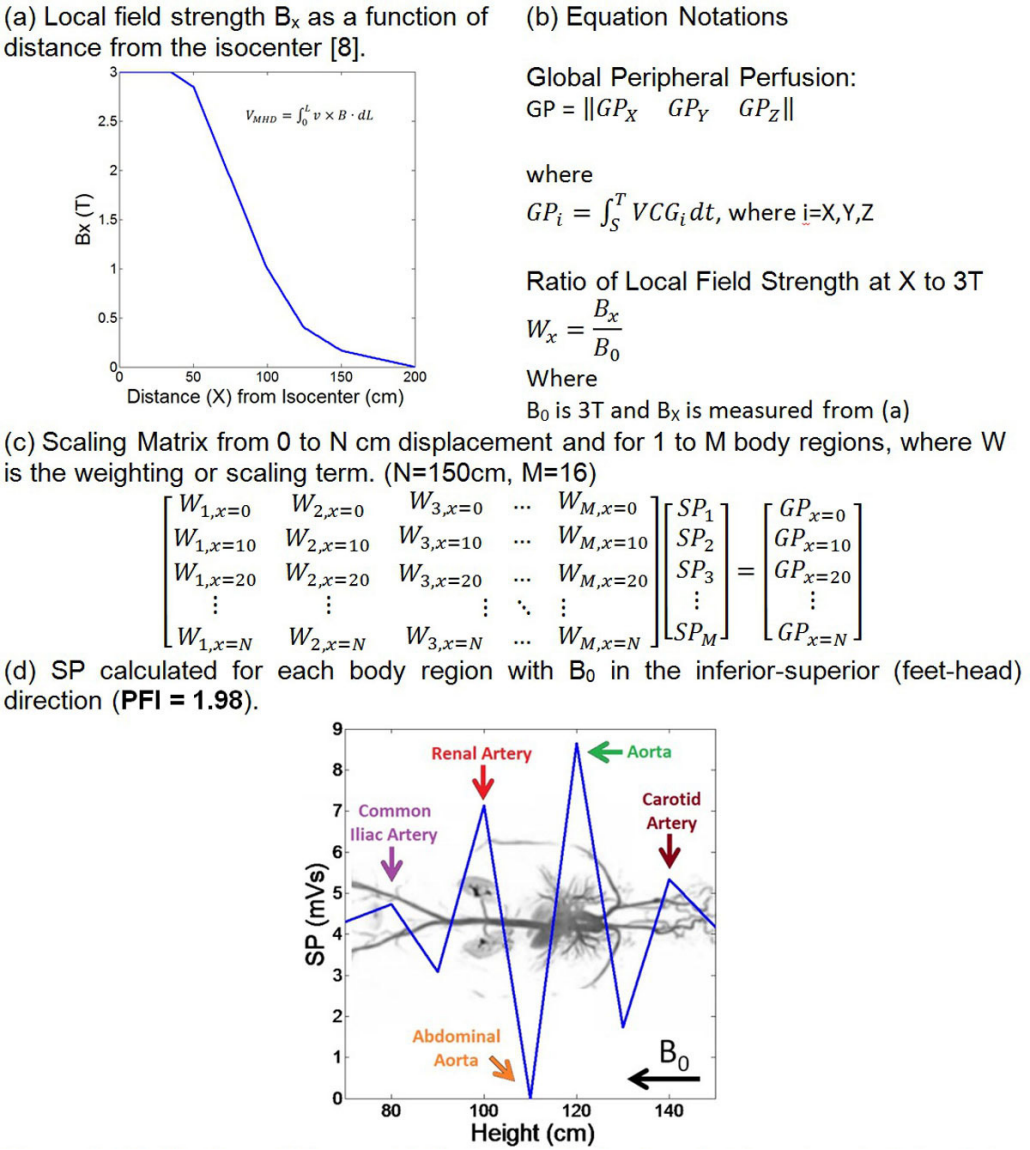
**Distribution of segmental peripheral perfusion at different part of the body overlaid with an MRI angiography **[9]**showing major vasculature in a human body**.

A GE digital-IT ECG recording system modified to be MRI-compatible [5] was used to record the 12-lead ECG of a volunteer subject at 3T. The subject was moved in 10-cm increments from the scanner fringe fields, 150 cm from the isocenter, until the heart was positioned at the isocenter (Figure 1). 12-lead ECG traces were converted into Vectorcardiograms (VCG) using an inverse Dower transform [6], VMHD vectors were extracted through subtraction of VCGs obtained in and outside the MRI [5], and time-integration of VMHD over the S-T segment was performed as a beat-to-beat metric for a Global Peripheral Perfusion (GP) index [5]. The GP metric is attributed to the Segmental Peripheral Perfusion (SP) of different body segments under varying magnetic field strength (BX); therefore a linear decomposition matrix, was applied to resolve the SP metric (Figure 2a-c). Reported SP values at different body segments were scaled to 3T for comparison (Figure 2d), and PFI was computed as the ratio of aortic and extremity SP [4,7].

## Results

SP varied over different body segments, with major blood vessels corresponding to greater changes in SP (Figure 1d). Fluctuations in SP were observed at the thigh-hip complex, kidneys, aorta, and head, which were attributed to the common iliac, renal, aortic, and carotid arteries, respectively. When the direction of the blood flow aligned with B0, SP was minimized, such as in the case of the abdominal aorta (Figure 1d). PFI was determined to be 1.98, within the normal range of 1.18-2.5 [1].

## Conclusions

VMHD processing using this method exhibits characteristic SP patterns and perfusion levels for each body segment. Measured PFI levels were comparable to normal values. Future work includes comparison of the processing result with paired PO-based PFI measurements.

## Funding

NIH U41-RR019703, NIH R03 EB013873-01A1, SBIR-1 R43 HL110427-01.
